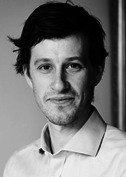# Electrode Materials in Modern Organic Electrochemistry

**DOI:** 10.1002/anie.202005745

**Published:** 2020-08-24

**Authors:** David M. Heard, Alastair J. J. Lennox

**Affiliations:** ^1^ University of Bristol School of Chemistry Cantocks Close Bristol, Avon BS8 1TS UK

**Keywords:** electrocatalysis, electrochemistry, electrode, materials, organic synthesis

## Abstract

The choice of electrode material is critical for achieving optimal yields and selectivity in synthetic organic electrochemistry. The material imparts significant influence on the kinetics and thermodynamics of electron transfer, and frequently defines the success or failure of a transformation. Electrode processes are complex and so the choice of a material is often empirical and the underlying mechanisms and rationale for success are unknown. In this review, we aim to highlight recent instances of electrode choice where rationale is offered, which should aid future reaction development.

## Graphical abstract

**The choice of electrode material** is critical for achieving optimal yields and selectivity in synthetic organic electrochemistry. The material imparts significant influence on the kinetics and thermodynamics of electron transfer. However, electrode processes are complex and so their optimisation is often empirical. Highlighted herein are recent instances of electrode choice where rationale is offered.

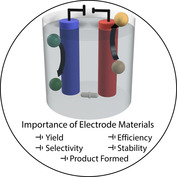

## 1 Introduction

With high control of the rate, location and driving force of electron‐transfer processes, electrochemistry is uniquely positioned to provide selectivity and sustainability benefits for the preparation of organic compounds. As these opportunities are being realised by academic and industrial research groups worldwide, the field of synthetic organic electrochemistry has received renewed interest over the last 5–10 years.^1^, ^2^, ^3^, ^4^, ^5^ New synthetic methodology and reactivity has been developed, including processes that are more inexpensive, safer and produce less waste than “classical” approaches.^6^, ^7^, ^8^, ^9^, ^10^, ^11^, ^12^ In addition, the relative ease with which the technique can be scaled is demonstrated by the fact that several industrial organic electrochemical processes have been developed.^13^, ^14^, ^15^, ^16^, ^17^, ^18^, ^19^

As the electron transfer between electrode and solution‐phase electrolyte is heterogenous, the development of synthetic organic electrochemical reactions requires close attention to parameters that are not traditionally encountered by organic chemists. As well as optimising the applied current density or potential difference across a cell, electrochemical reactions can be performed in either batch or flow cells, and divided or undivided cells. However, it is the electrodes that constitute the most important difference, as the success or selectivity of a particular transformation is highly dependent on the material. Not only does the electrode material itself determine the mechanism of electron transfer, but the electrode separation distance, shape and size determine the submerged surface area, the field homogeneity and the resulting current density; all of which can affect the reaction outcome. While the electrode material is an additional parameter that requires optimisation, it can be exploited to control and change the selectivity of a reaction, and provides opportunities to vary reactivity through electrode‐catalysis, (electrocatalysis), mediator‐modified or chemically‐modified electrocatalysis.

**Figure 1:**
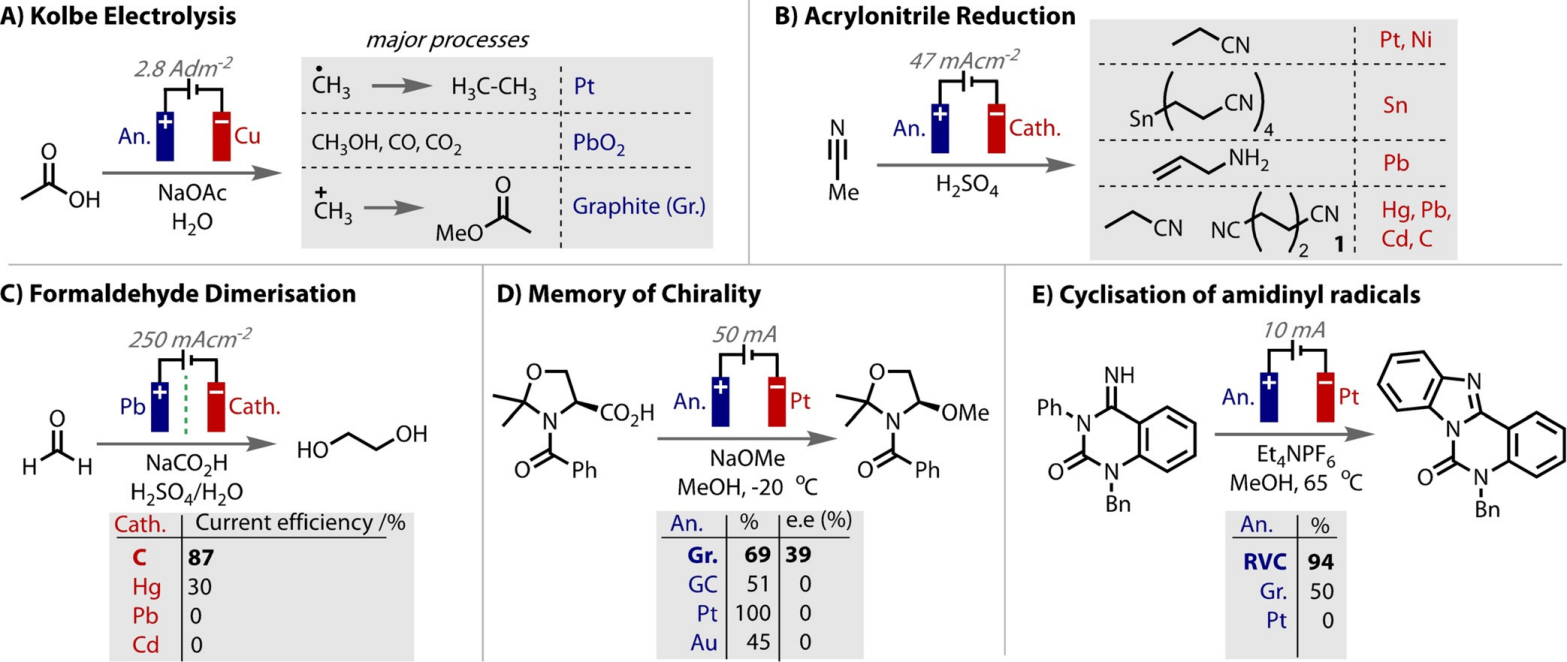
Examples of the effect of electrode materials on classic (A–D) and contemporary (E) reactions. Gr.=graphite, GC=glassy carbon; RVC=reticulated vitreous carbon.

The ability of specific materials to give unique outcomes and determine the selectivity for synthetic electrochemical reactions has long been recognised.^20^, ^21^ Classical examples include the anodic oxidation of acetic acid in aqueous solutions (Figure 1 A), in which the identity of the products and their distribution varies with different anode materials,^22^, ^23^, ^24^, ^25^ and the reduction of acrylonitrile in which the reaction products strongly depend on the cathode material (Figure 1 B).^26^, ^27^, ^28^ In this reaction, the formation of adiponitrile (**1**) with cadmium and steel electrodes is a mega‐tonne per annum industrial process^15^ that is used in the production of nylon‐6,6, thus exemplifying the importance of the control that the electrode material imparts, and the possible ramifications of its variation. The choice of electrode material can impart a more binary outcome by switching reactivity on or off. Classic examples of this include the cathodic hydrodimerization of formaldehyde to ethylene glycol, wherein product is only obtained with the use of mercury or carbon cathodes and no product is observed with lead or cadmium (Figure 1 C).^29^ In addition, memory of chirality (enantiomeric excess) was only observed with the use of graphite anodes in a decarboxylative etherification reaction (Figure 1 D).^30^ In a more recent example, Xu reports a drastic change in yield when exploring the electrode material in an aromatic C−H functionalisation reaction with electrochemically‐generated amidinyl radicals (Figure 1 E).^31^ Varying the electrolyte or the applied current had a relatively minor effect on the yield of the reaction, but replacing the reticulated vitreous carbon (RVC) anode with Pt completely shut down reactivity, whereas graphite gave an intermediate outcome.

While the differences in the outcomes of these reactions with different electrode materials are stark, the high complexity of electrode processes commonly renders the generation of conclusive explanations very difficult. Indeed, it has been noted elsewhere that it is “impossible to select the optimum electrode for a given process on a theoretical basis. Instead, an empirical approach must be used”.^21^ While it is true that an empirical approach is currently the most efficient strategy to optimise a reaction, an appreciation of the influence of electrode materials and a greater understanding of electrode processes should lead to a more informed approach and new opportunities in the field. In addition to this, poor reproducibility is a major challenge that accompanies the use of electrochemistry in organic synthesis, and differences in the electrode material, grade and source all contribute to this problem. Thus, an appreciation of the important factors associated with the electrode should facilitate an improved rationalisation of the differences between reported and achieved yields or selectivity.

In this review, we initially summarise the most important practical and reactivity considerations for electrode materials in organic electrochemistry. Then, our goal is to highlight examples in which the performance of a particular electrode material is found to be unique and important. We place an emphasis on contributions to the literature from the last decade, while focussing on synthetic organic transformations and practical considerations on regular laboratory scales. It should be noted that although an explanation on electrode choice is given in an increasing number of cases, of the protocols that we have surveyed from the last decade, only a small percentage (ca. <5 %) provide some supporting insight. Reaction selectivity and yields can also very much depend on other reaction parameters, and so it is not always clear if the electrode material itself exclusively defines the observed difference. As this point adds to the ambiguity, the examples have been selected as carefully as possible, in preference to providing an exhaustive coverage. Thus, the reader is referred to a number of earlier review articles that are also relevant to these themes.^21^, ^32^, ^33^, ^34^, ^35^, ^36^, ^37^, ^38^, ^39^, ^40^ Beyond the scope of this review are photoelectrodes,^41^, ^42^, ^43^, ^44^, ^45^, ^46^, ^47^ and other practical aspects of electrochemistry, which have been addressed in recent tutorial reviews.^48^, ^49^, ^50^, ^51^, ^52^, ^53^, ^54^

## 2 Electrode Selection

### 2.1 Practical Aspects

The primary judgement of candidate materials will be based on their performance in the reaction, i.e., yields and selectivity, but current efficiencies, obvious signs of corrosion, cost, availability and machinability are other critical factors, the relative importance of which will vary according to the specific process. In other applications of electrochemistry‐such as those focussed on energy or bulk scale commodity production‐small, single digit differences of efficiency gains can be extremely critical, for example from the use of a precise grade of graphite. However, in organic synthesis where the scales are comparatively smaller, larger gains in yield or complete switches in selectivity become more important. This is because the cost of the electrode material and the man‐hours that are required to optimise a process must be balanced against the costs of the reagents and the value of the product. For example, as the price of electricity is typically low compared to the contents of a reaction mixture, achieving small gains in current efficiency is not the highest priority for reaction optimisation. This will only become a consideration if the scale is increased and the value of product is lowered.

Whilst electrodes can in principal be made from any conductive material, in order to make an appropriate choice there are a number of mechanical and electrochemical properties to consider. An idealised electrode material should be inexpensive, non‐toxic, stable to a wide range of temperatures, pressures and solvents, yet able to be manipulated into forms for electrode construction, such as rods, wires, plates, foams and meshes. Most electrodes consist of a single material, but a support material combined with an electroactive coating, such as Pt, can also be used.^20^ In organic solvents, which are more resistive than aqueous systems, the use of 3‐dimensional, high surface area electrodes is advantageous, as they impart decreased current density and cell potential. Thus, the use of RVC or carbon felt can increase productivity as higher currents can be applied.^37^, ^55^ Between electrode materials, surface area can vary dramatically, for example, the surface area of a “smooth electrode” can be up to 3 orders of magnitude lower than a porous surface, such as platinised platinum.^56^

An electrode should be stable and resist corrosion. An exception to this is when the electrode is sacrificial, for example when metal ionisation is intended as a counter electrode process, or when the metal ions are used to stabilise a product, such as in a carboxylation reaction.^57^ Degradation of electrodes by mechanical action can occur as a consequence of convection forces in the reaction vessel, such as the release of graphite particulates, which requires separation via filtration. In addition, fragile materials, such as low pore density RVC, can lead to difficulties in physical handling and manipulation. Swelling of the electrode can also be problematic with certain electrode material/electrolyte combinations.

The use of electrodes with high resistivity leads to an ohmic (IR) drop, which creates the requirement for a higher cell potential. This excess energy input is likely lost as heat into the reaction medium, which is inefficient and may be deleterious to the reaction outcome.^58^ On an industrial scale, this can limit the choice of materials to only those that are highly conductive, or require special electrode architectures.^21^ Once a material is formed into an electrode, a low ohmic resistance connection should also be made.

### 2.2 Reactivity Aspects

**Figure 2:**
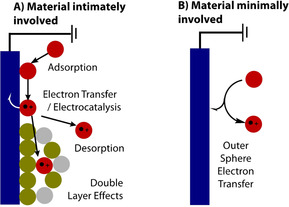
The two limiting cases for electron‐transfer.

The mechanism for electron transfer at an electrode occurs between two limiting scenarios. In the first limiting case (Figure 2 A), the electrode surface is intimately involved in the mechanism of electron transfer and acts as a catalyst in the reaction; i.e., electrocatalysis.^52^ The products, mechanism and kinetics of electrode reaction in this case are highly dependent on the composition of the electrode material, meaning that small differences may be extremely significant in determining the outcome of the reaction. Conversely, in the second limiting case (Figure 2 B), the electrode is completely inert and provides a source or sink of electrons that are transferred in an outer‐sphere manner between the substrate and electrode. The identity of the products formed, and the mechanism and kinetics of their formation should be independent of the material.

The potential required beyond that necessitated by thermodynamics to drive a reaction at a practical rate is referred to as the *overpotential* (η).^59^ The observed overpotential in a particular system is a sum of the individual overpotentials for each step in the process, such as adsorption, charge‐transfer, desorption and mass‐transport (diffusion, convection and migration) overpotentials. As the electrode material dictates the mechanism of electron transfer, it is the biggest contributing factor to the overall overpotential of a process. This important factor will be responsible for outcome variations observed during reaction optimisation.

**Figure 3:**
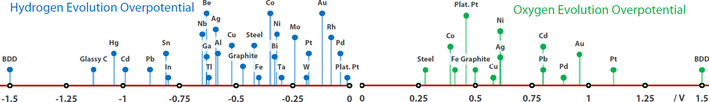
HER and OER overpotentials taken from Table 1 (averaged where relevant) for various electrode materials.

**Figure 4:**
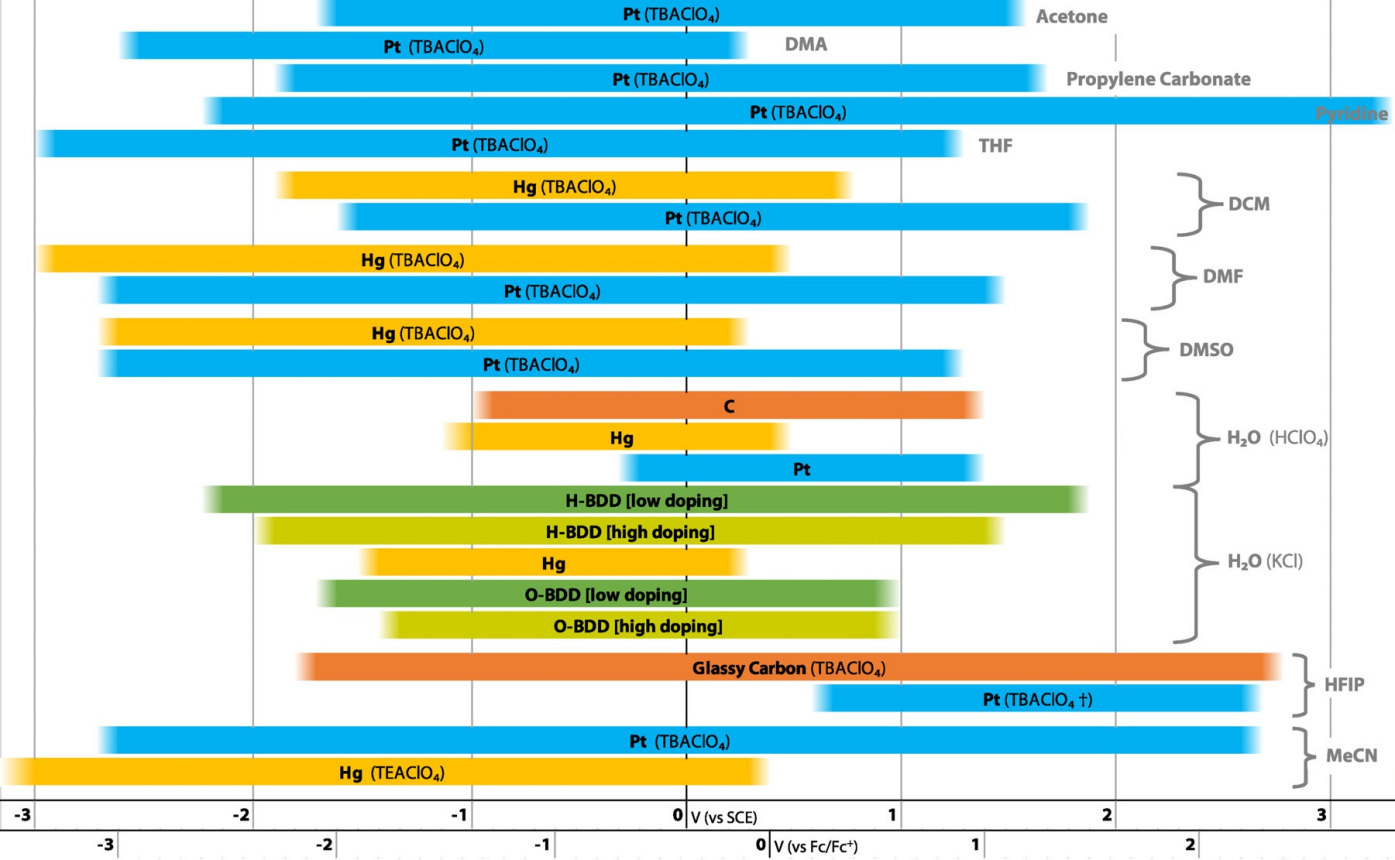
Solvent windows various electrode material and electrolyte combinations.^33^, ^123^, ^124^, ^125^, ^126^, ^127^, ^128^, ^129^ († current density cut‐off at *j*=±0.1 mA cm^−2^.).

For many reactions, such as the Hydrogen Evolution Reaction (HER) or Oxygen Evolution Reaction (OER), the decrease in overpotential through new electrode materials is the subject of intense investigation.^60^, ^61^, ^62^, ^63^, ^64^, ^65^, ^66^, ^67^, ^68^, ^69^ Small efficiency gains will translate into large cost savings when these processes are conducted on scale. However, of greater importance to synthetic organic electrochemistry is the selectivity changes or suppression of side reactions that are enabled by the different overpotentials for each process on different electrode materials. An example of this control in a substrate‐reduction reaction is to suppress competing proton reduction (HER) by choosing a cathode material that has a high overpotential for this process. Indeed, the overpotentials on common electrode materials varies considerably for the HER and OER, Table 1 and Figure 3. A low overpotential for the desired redox reaction will not only ensure the reaction can be driven more efficiently but will improve selectivity over competing processes. The overpotential for solvent oxidation or reduction can also vary significantly on different electrode materials.^70^ This variation has implications for the width of the potential solvent window that is available to a reaction and therefore to the extent of redox chemistry that can be performed, Figure 4.

**Table 1:** Overpotentials (HER and OER) and conductivities for various electrode materials.

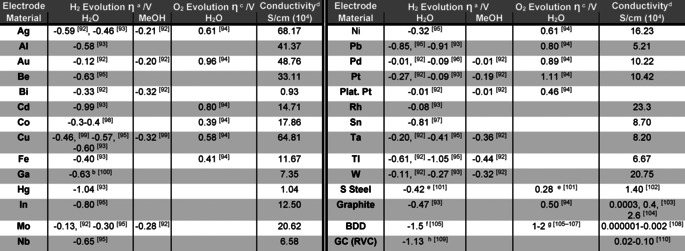

Overpotential is also influenced by supporting electrolyte,^93^ solvent,^99^, ^111^, ^112^, ^113^ current density,^95^, ^96^ concentration,^114^ temperature,^115^ and any additives,^21^, ^116^, ^117^, ^118^, ^119^, which will all affect the direct application of these figures. ^a^ Hydrogen evolution (HER) overpotential recorded at 1 mA cm^−2^, 25 °C, 1 m HCl (or H_2_SO_4_) in the solvent specified, unless otherwise indicated. HER data for various alloys (not listed) also available.^120^ ^b^ recorded at 2×10^−4^ A cm^−2^. ^c^ Oxygen evolution (OER) overpotential recorded at 1 mA cm^−2^, 25 °C, 1M KOH in water, unless otherwise indicated. ^d^ Recorded at 273 K (except Hg, glassy carbon, BDD that were recorded at 298 K), data taken from ref. 121, 122 except where specifically given. ^e^ 1 m KOH. ^f^ 0.5 m H_2_SO_4_. ^g^ Highly dependent on doping and treatment. ^h^ pH 3.4.

**Figure 5:**
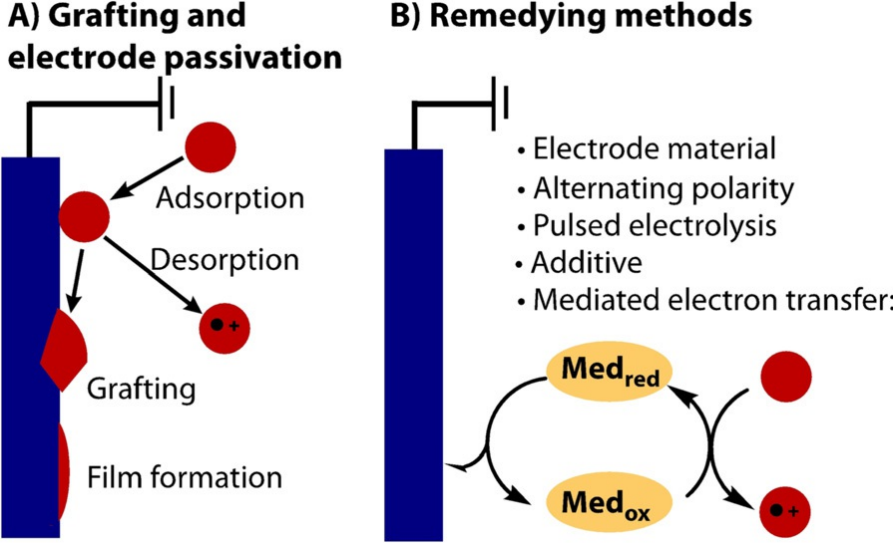
Electrode passivation.

The stability of an electrode is important for ensuring longevity of use. However, the stability of the substrate or the intermediates produced on the electrode is also important for ensuring high yields of product. A compound can irreversibly bind and decompose on the surface, leading to a decreased mass balance and yield of product (Figure 5 A). Strategies for grafting organic compounds onto electrode surfaces for intentional surface modification have also been reported.^71^, ^72^, ^73^, ^74^ However, unintentional grafting can vary in degree depending on the specific redox event, electrolyte and electrode material. The result is a passivated electrode with decreased electrode activity due to the formation of an electrically insulating layer. Electrode passivation can be detected by cycling a cyclic voltammetry (CV) experiment and observing the current decrease with each cycle,^75^, ^76^, ^77^ with the current not being restored to its original value until the electrode is cleaned. Examples of electrode passivation are tight oxide films on metals that are formed at high anodic potentials,^78^, ^79^ insoluble oxidation products, polymer deposits generated by anodic oxidation of olefinic, aromatic or phenolic compounds,^80^, ^81^, ^82^ or solutions of HF or ionic liquids.^83^, ^84^ Optimisation of the electrode material is a key task in remedying this effect (Figure 5 B). Other methods that have been shown to be effective include pulse electrolysis,^85^ sonication,^86^ alternating the polarity of the electrodes (which can also effect the reaction selectivity or yield),^87^, ^88^, ^89^ and the use of mediators to shuttle redox equivalents from the electrode to the substrate in the bulk phase.^75^, ^90^ Alternatively, the addition of additives can increase the solubility of the insulating polymer in the electrolyte or protect the electrode surface, which has been shown to be highly effective in a recent electrochemical Birch reduction.^91^

### 2.3 Trends

**Figure 6:**
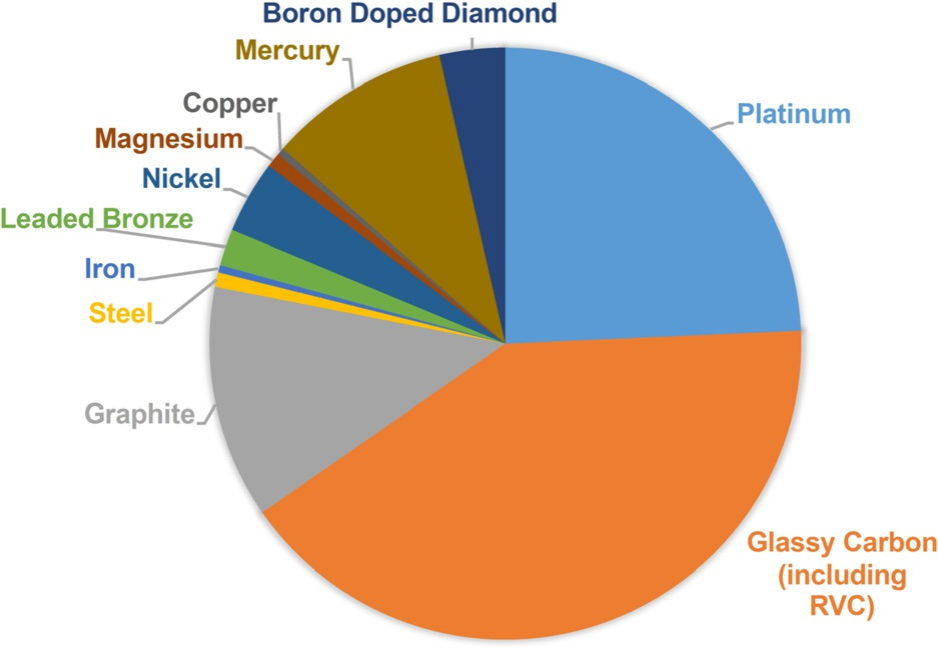
Occurrence of electrode materials used (cathode or anode) in a survey of 915 synthetic electrochemical protocols published between 2000–2017.

The factors that contribute to the choice of electrode material vary and can be very specific. The number of electrode materials available has increased over time and trends of use have changed and evolved. For example, lead and mercury were previously preferred due to their high hydrogen overpotential (η_H_) and stability to acidic media. With mercury being in the liquid state, the surface is constantly renewed and can remain clean and free of impurities. However, concern over the high toxicity of these metals has limited more recent wide‐spread use and hence other materials have attracted greater attention. Modern organic electrochemical methodology relies more heavily on platinum, which is robust, easy to clean and redox stable, as well as carbon‐based electrodes that are more inexpensive and thus appropriate when the scale of a reaction renders the cost of platinum prohibitive (Figure 6).^130^, ^131^ Glassy carbon is the most commonly used carbon material, which is the fullerene allotrope of carbon,^132^ and includes the high‐surface area foam form, RVC.^34^ Graphite is also a commonly used form of carbon electrode, which is less chemically inert than glassy carbon but more conductive^133^ and is less expensive. The diamond allotrope of carbon can also be used, Boron Doped Diamond (BDD) has emerged as a unique material and is becoming increasingly popular.^134^, ^135^, ^136^, ^137^ There has also been evidence for the emergence of new materials, metals or alloys used as electrodes in organic synthesis, such as leaded bronze, tantalum, niobium or molybdenum.^138^, ^139^, ^140^, ^141^ No doubt this trend will continue as the electrode processes with each material become better understood, wider range of materials are adopted, and the further development of idealised materials.

## 3 Electrocatalysis: Specific Adsorption and Surface Interactions

At the extremity of the first limiting case (Figure 2), the electrode surface is explicitly involved in the reaction mechanism through specific adsorption and surface interactions. As well as providing the required redox equivalents, the electrode surface serves to catalyse the reaction, and is thus known as *electrocatalysis*. Savéant defined “Electrocatalysis” as the term to “*name catalysis of electrochemical reactions by surface states of the electrode*”,^52^ which is distinct from mediated electrolysis that employs molecularly defined catalysts. The precise nature of these interactions varies, depends on each specific reaction and can often be the subject of much debate. Nevertheless, theoretical models are improving and can now describe catalytic reactions in great detail.^142^ The strength of interaction (adsorption vs. desorption), the timing and order of electron transfers and the concertedness of steps are all relevant when considering the mechanism. Adsorption describes a variety of more specific interactions of a substrate with the electrode, such as strong electrostatic interactions, π‐interactions and chemical bonds. As well as the smooth material itself, the sites of binding and catalysis may be impurities, edge or end atoms, deposited nanoparticles, thin films or single atoms of a secondary or different material to the bulk material.^143^, ^144^, ^145^

The strength of interaction between substrate and electrode should be strong enough to trigger a reaction, but not too strong that the product fails to dissociate and desorb. This balance is known as the Sabatier principle^146^ and has been shown to contribute to the bell curves observed for rates of electrocatalytic HER.^147^, ^148^ In organic electrochemistry, it is common for products to avoid dissociation from the electrode (Figure 5 A), which can lead to decomposition and a low mass balance at the end of reaction.

### 3.1 Working Electrode Material

**Figure 7:**
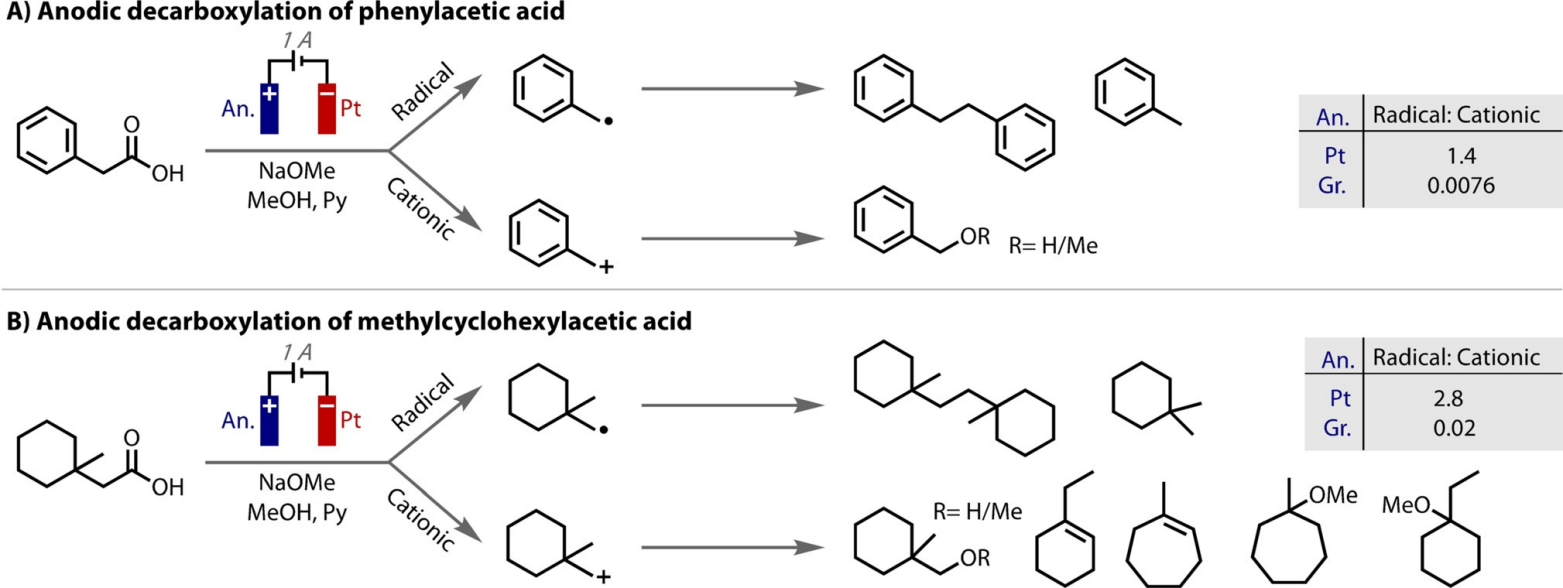
Product distributions arising from anodic decarboxylation on either platinum or graphite electrodes.

In a classical example, the extent of electrocatalysis in the oxidative decarboxylative Kolbe and Hofer–Moest reactions has been the subject of much debate in the literature over the years.^149^, ^150^, ^151^, ^152^ In one study, the product distributions from the use of a platinum and carbon anodes were compared. It was found that the ratio of 1‐electron vs. 2‐electron oxidation products (i.e., ratio of products‐from‐radicals over products‐from‐cations) was much greater with platinum anodes than with graphite anodes (Figure 7).^153^ Carbon anodes are more efficient than platinum anodes at removing a second electron, to form a cation (with a proton loss).^145^ The difference was proposed to be due to a greater tendency of radicals to adsorb onto carbon because of the presence of paramagnetic centres in the material. Thus, the adsorbed radicals on carbon undergo further oxidation to form a carbocation that is then electrostatically repelled and primed to react with nucleophiles. However, the radicals produced on a platinum surface are largely desorbed and so participate in radical reactions. This effect has also been recorded in other transformations, such as in the electrochemical cyclisation of dienes, in which Moeller observes a difference in the efficiency of 1‐ vs. 2‐electron pathways when using platinum and carbon anodes.^154^

More recently, electrocatalysis has been especially noted for cathodic processes; it has even been remarked that *“it seems uncertain that totally inert electrodes exist […] within the cathodic range*”.^155^ In particular, the dehalogenation of aryl and alkyl‐halides with different cathodic materials has been the subject of significant investigation.^21^, ^155^, ^156^, ^157^ The over‐potential, concertedness and degree of interaction varies with different electrode materials and can lead to the formation of different product distributions. For example, the use of silver cathodes significantly decreases the over‐potential necessary to cleave a C−X bond,^157^ compared to mercury or glassy carbon cathodes. In the reduction of linear alkyliodides on smooth silver cathodes, there is evidence for the transient formation of [Ag^+^−R] I^−^ species on the interface.^155^ The formation of such species will stabilise the radical and ensure a lower reduction overpotential. Compared with glassy carbon electrodes, copper has also been found to show exceptional electrocatalytic properties, either as a smooth metal or when deposited onto a conducting surface.^158^

**Figure 8:**
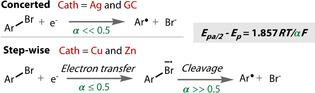
Mechanistic elucidation of reductive debromination on different cathode materials.

The electrocatalytic dehalogenation of aryl‐halides can occur via a stepwise electron transfer‐cleavage mechanism, or a concerted process. Recent analysis of an extensive range of cathode materials revealed a strong dependency of the mechanism of debromination on the electrode material (Figure 8).^159^ Electron transfer coefficients (α) give an indication of how reactant or product‐like the transition state is in terms of its electrical behaviour. These were extracted from CVs by analysis of the difference between the peak potential and half‐wave potential, and used as an indication for the mechanism. A value of α significantly lower than 0.5 indicates a concerted mechanism, whereas a stepwise mechanism will either have an α significantly higher than 0.5 if cleavage is the RDS or only slightly lower than 0.5 if ET is the RDS. Thus, reduction potentials and electron transfer coefficients were measured by cyclic voltammetry for the reduction of different aromatic bromides on different electrode materials. Only 4 of the 11 materials showed reduction features in the CV. Ag and glassy carbon were found to both follow a concerted mechanism: Ag exhibited a remarkable electrocatalytic activity with a 0.9 V lower overpotential than glassy carbon. This overpotential difference is a considerable thermodynamic improvement and demonstrates the significant effect that materials can have on the overpotential of an electrochemical redox event. Cu and Zn electrodes were found to give step‐wise mechanisms, with rate determining ET and cleavage steps, respectively.

The cathode material can determine the distribution of products from a reaction. The reduction products of alkylhalides have been reported by Peters to vary according to the cathode material.^160^, ^161^ On vitreous carbon cathodes, *n*‐decane and 1‐decene are yielded from the reduction of iododecane, whilst on a silver cathode, a dimeric product was formed. When testing secondary alkyl halides, it was interesting to note that the product distribution switched, such that dimers were predominately formed at carbon‐based cathodes.

**Figure 9:**
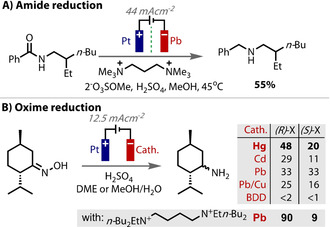
A,B) Cathode material with a high overpotential for proton reduction is necessary for attaining good selectivity for substrate reduction.

Avoiding the adsorption of reagents and the subsequent electrocatalysis of competing side‐reactions can be critical to the success of a desired reaction. This can be achieved using an electrode material with a high overpotential for the competing processes. For example, in a reduction reaction, competing proton reduction can be avoided through the use of a cathode with a high overpotential for that process, such as glassy carbon, mercury or lead. Lead has found use for this reason in the deoxygenation of amides and sulfoxides (Figure 9 A).^162^ Amides are thermodynamically difficult to reduce and the presence of acid is necessary to provide the equivalents of protons. Therefore, it was important to use a material that preferentially reduces amides over protons, and lead was found to be superior for that.

In the reduction of menthone oximes to menthylamines (Figure 9 B), Waldvogel screened cathode materials to avoid any competing proton reduction.^119^, ^163^ Those with only a moderate hydrogen overpotential, such as titanium, iron, copper, zinc, indium, tin and bismuth, all failed to produce the desired product, and so it was necessary to use a high overpotential material, such as lead or mercury. The electrode material also influenced the selectivity of the reaction: whereas, mercury and cadmium cathodes led to pronounced diastereoselectivity, lead or copper/lead gave either no or little selectivity. The authors proposed that good diastereoselectivity was due to stabilisation of the reactive intermediate by stronger binding to the electrode surface and slowing down conformational switching.

**Figure 10:**
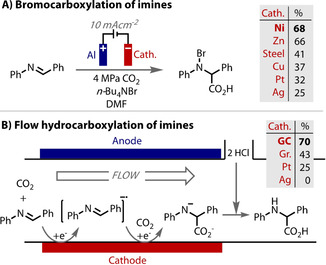
A) Increased adsorption of imine on Ag cathode leading to decomposition and dimerization avoided. B) Materials with high overpotential for CO_2_ reduction required for attaining highest product yields.

The efficiency of the electrochemical reductive carboxylation of imines to yield *N*‐bromoamino acids also depends on the cathode material (Figure 10 A).^164^ In this case, the yield was proposed to correlate with the strength of substrate adsorption onto the electrode surface. Silver was noted to exhibit pronounced specific adsorption of imines, which leads to a higher concentration of imines on the electrode surface, and therefore more facile decomposition and accelerated imine dimerisation. Highest yields were reported using nickel cathodes. When adapting the reaction into flow, Atobe considered cathode materials with a high overpotential for carbon dioxide reduction (Figure 10 B).^165^ In this example, the overpotentials (determined by linear sweep voltammetry) correlated strongly with yield. Glassy carbon gave the highest efficiency, followed by graphite, then platinum and lastly silver. Reduction of the imine to a radical anion is necessary for a productive reaction to take place. Any competing direct CO_2_ reduction decreases charge efficiency and reaction yields.

**Figure 11:**
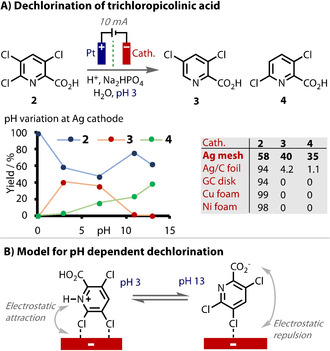
A) Unique electrocatalytic behaviour of Ag exploited for dechlorination of **2**. with trend at different pHs. B) Proposed pH dependent adsorption mode.

The electrochemical hydrodechlorination of 3,5,6‐trichloropicolinic acid (3,5,6‐T) is an example of a reaction in which the cathode material proved absolutely crucial to reaction success.^166^ Using copper, glassy carbon or nickel cathodes, the reaction was completely unsuccessful (Figure 11 A). Only the use of silver produced any desired 3,5‐dichloropicolinic acid (**3**) product, a compound with significant pharmaceutical relevance. It was proposed that the overpotential for proton reduction was lower with the use of Cu and Ni, making substrate reduction more difficult. The productivity of silver cathodes compared to glassy carbon (GC) cathodes was ascribed to an electrocatalytic effect of silver that was not possible on carbon (Figure 11 B). Interestingly, the selectivity of hydrodechlorination was found to be dependent on the pH as well. Electrostatic forces engendered high selectivity for the 3,5‐substituted isomer (**3**) at pH 3, and the 3,6‐substituted isomer (**4**) at pH 13 (97 %) (Figure 11 B).

### 3.2 Counter Electrode Material: Electrogenerated Base

**Figure 12:**
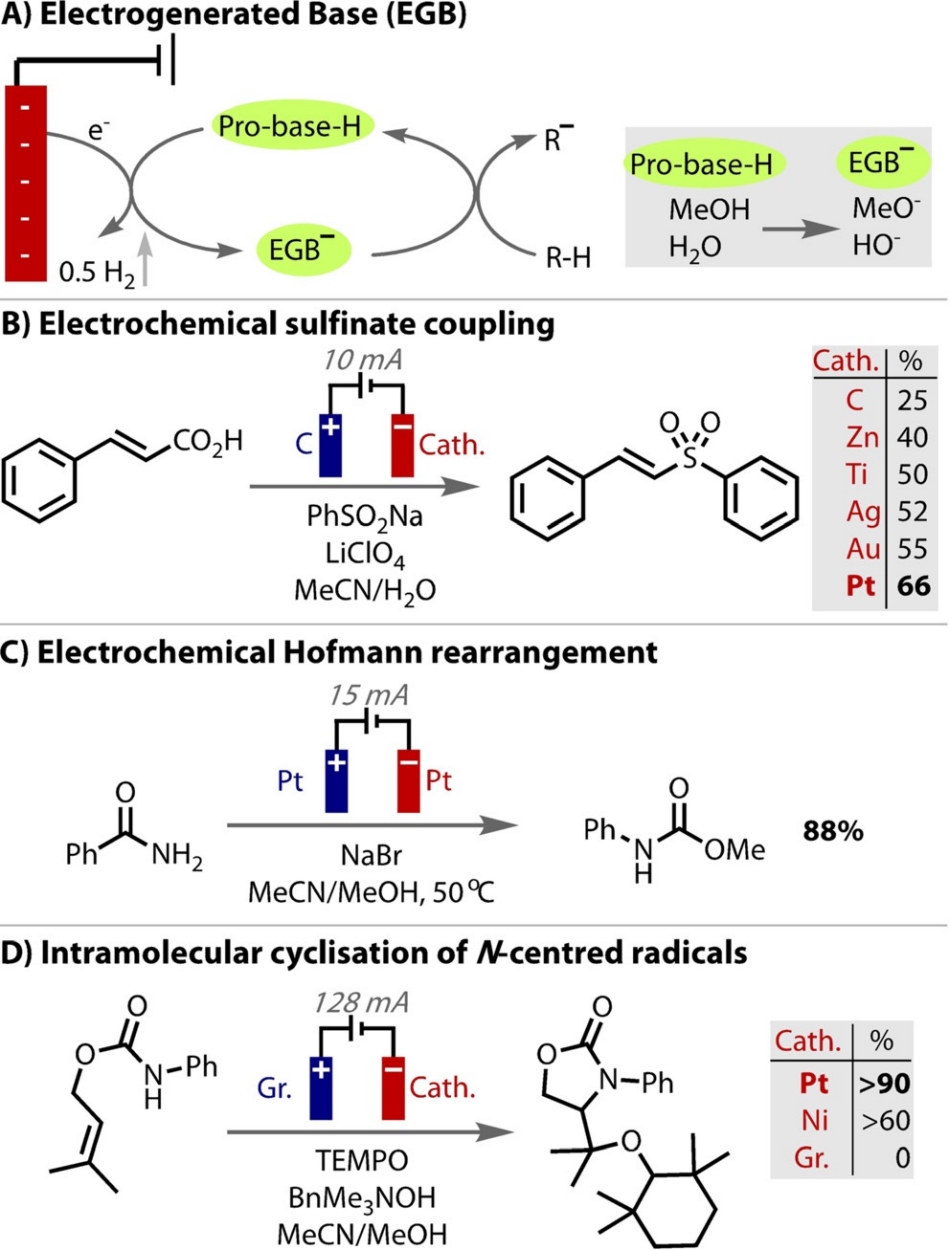
A) Generation of a base on the cathode. B–D) A low overpotential for proton reduction on the counter electrode renders a milder generation of a base, which improves product yields.

As well as tuning the overpotential on the working electrode, the overpotential on the counter electrode reaction is also important to consider and has often been shown to be key to the success of a reaction. Of particularly frequent consequence is the reduction of protons to evolve hydrogen (HER) at the cathode to form a base. The concept of forming electrogenerated bases (EGBs, Figure 12 A)^167^, ^168^ in situ from a pro‐base for utilisation in a synthetic transformation was first reported in 1967.^169^ Using electrochemistry allows the concentration and basicity of the reaction to be carefully controlled,^170^ and changing the counterion influences both the stability and reactivity of the EGB.^171^

To generate bases electrochemically (EGB), the reduction potential of the pro‐base must be less negative than any other species in the reaction (including the product), which renders the overpotential for proton reduction vital for success to be achieved. By promoting hydrogen evolution over other potential reductive processes, the choice of cathode material influences the outcome of anodic transformations.^138^, ^172^, ^173^ An example of this is in the synthesis of (*E*)‐vinylsulfones from cinnamic acids, in which Wang found a significant dependence of the reaction on the counter electrode material (Figure 12 B).^174^ Whilst the reaction is oxidative with respect to the substrates, the cathodic generation of base is required for the deprotonation of the carboxylic acid. Platinum was the best performing cathode material, which has the lowest overpotential for proton reduction, whilst glassy carbon, which has a higher overpotential, resulted in a decreased yield. Materials with a medium η_H_ performed in‐between these two cases. Thus, a lower potential difference is necessary with Pt under constant current electrolysis conditions, which limits competing reduction processes. Similarly, Zhang switched from carbon to platinum counter electrodes in an electrochemical Hofmann rearrangement, to more readily form methoxide on the cathode and found that yields improved (Figure 12 C).^175^

The electrochemical oxidative formation of *N*‐centred radicals and their intramolecular cyclisation onto alkenes has been developed by Moeller and Xu.^176^, ^177^, ^178^, ^179^, ^180^, ^181^, ^182^ Electrode materials were thoroughly analysed by Wirth when the process was transferred to a flow cell set up.^183^ Interestingly, it was found that the choice of counter electrode material had a stronger influence on yield than the choice of the working electrode material. A yield of over 90 % was achieved with a platinum cathode, around 60 % with nickel, and no reaction occurred with a graphite cathode (Figure 12 D). These results directly correlate with the over‐potentials for proton reduction (Pt=−0.09 V; Ni=−0.32 V; graphite=−0.47 V, Table 1). Yields were consistent (ca. <5 %) between the anodic materials tested with a Pt cathode. Correct choice of cathode material therefore meant the anode material could be chosen by cost, rather than due to its influence on the reaction.

**Figure 13:**
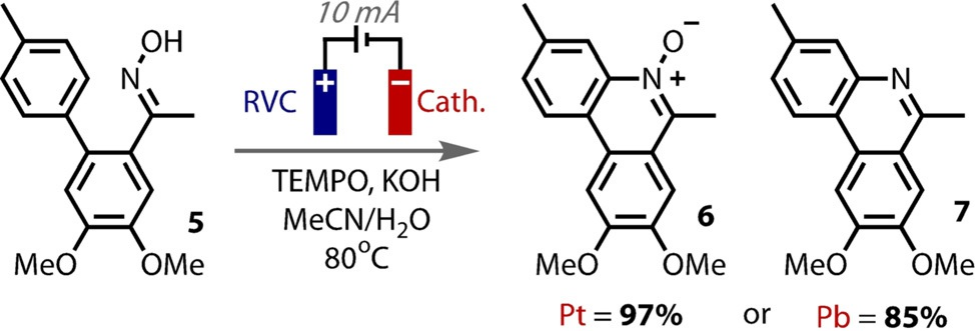
Low overpotential for proton reduction on Pt counter electrode gives **6**, but high overpotential on Pb means **6** is preferentially reduced to give **7**.

In an elegant example of the importance of counter electrode material, Xu recently reported a complete selectivity switch when the cathode material was switched from platinum to lead.^184^ The reaction is an oxidative TEMPO catalyzed intramolecular arene amination from oxime **5** (Figure 13). When platinum was used as the counter electrode, the low overpotential for proton reduction resulted in the *N*‐oxide product **6** remaining intact. However, when lead was employed, the deoxygenated heterocycle **7** was returned. This is because lead has a much higher hydrogen overpotential for proton reduction, meaning proton reduction is more difficult and so *N*‐oxide **6** now preferentially reduced on the counter electrode.

### 3.3 Modified Electrode Surfaces

The modification of electrode surfaces to aid catalysis of a reaction and decrease the overpotential for electron transfer is a technique that is well established, especially for energy‐related applications, such as the HER, OER and CO_2_ reduction.^60^, ^61^, ^62^, ^63^, ^64^, ^65^, ^66^, ^67^, ^68^ For synthetic applications, the immobilisation or tethering of electron transfer mediators onto electrode surfaces, either covalently^185^ or non‐covalently^186^ has been shown to improve the efficiency of reactions. Carbon electrodes are especially effective supports for catalysts as they can be readily functionalised.^74^ For example, oxidation produces a high density of surface carboxyl groups with which amide bonds can be formed, or the single electron reduction of diazonium cations reveals arene radicals that readily combine with graphitic electrodes.^187^, ^188^ Further details of the mediator‐immobilisation approach are, however, beyond the scope of this review, and the interested reader is directed to other relevant reviews.^74^, ^189^

**Figure 14:**
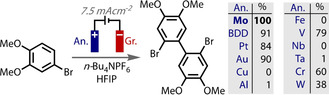
Surface modification through in situ generation of active Mo^V^ coating on anode.

The bulk surface modification of electrodes through, for example, polymer coating or nanoparticle deposition is comparatively less well exploited for organic synthesis compared to energy applications.^190^ A recent example demonstrated that the in situ generation of an active Mo^V^ layer on the surface of a Mo anode was responsible for greatly enhanced yields in the dehydrogenative coupling of arenes (Figure 14).^138^ Whilst this arene coupling reaction could be performed with BDD, Pt, Au, V, Cr or W electrodes, the efficiency was less optimal than with the use of Mo. Only very low levels of molybdenum were detected by mass spectrometry in the electrolyte solution, which is evidence that the active Mo^V^ species is only present on the surface and not released into solution.

## 4 Double Layer Control

Upon application of a potential to an electrode in solution, an ordering process occurs to form a structure of oppositely charged ions and solvent molecules at the surface, commonly known as the Helmholtz double layer. There have been several theoretical models proposed for this interfacial region (Helmholtz, Gouy–Chapman, Stern) but the precise behaviour depends on the nature of electrode (material and surface properties), as well as the composition of the electrolyte (supporting electrolyte, solvent).^21^ Unlike under aqueous conditions, the structure and thickness of the double layer in organic solvents is not well‐understood. Nevertheless, the structure determines the potential distribution close to the surface and the uniformity of current. The double layer thus influences the local driving force for electron transfer, which determines the kinetics of electron transfer.

**Figure 15:**
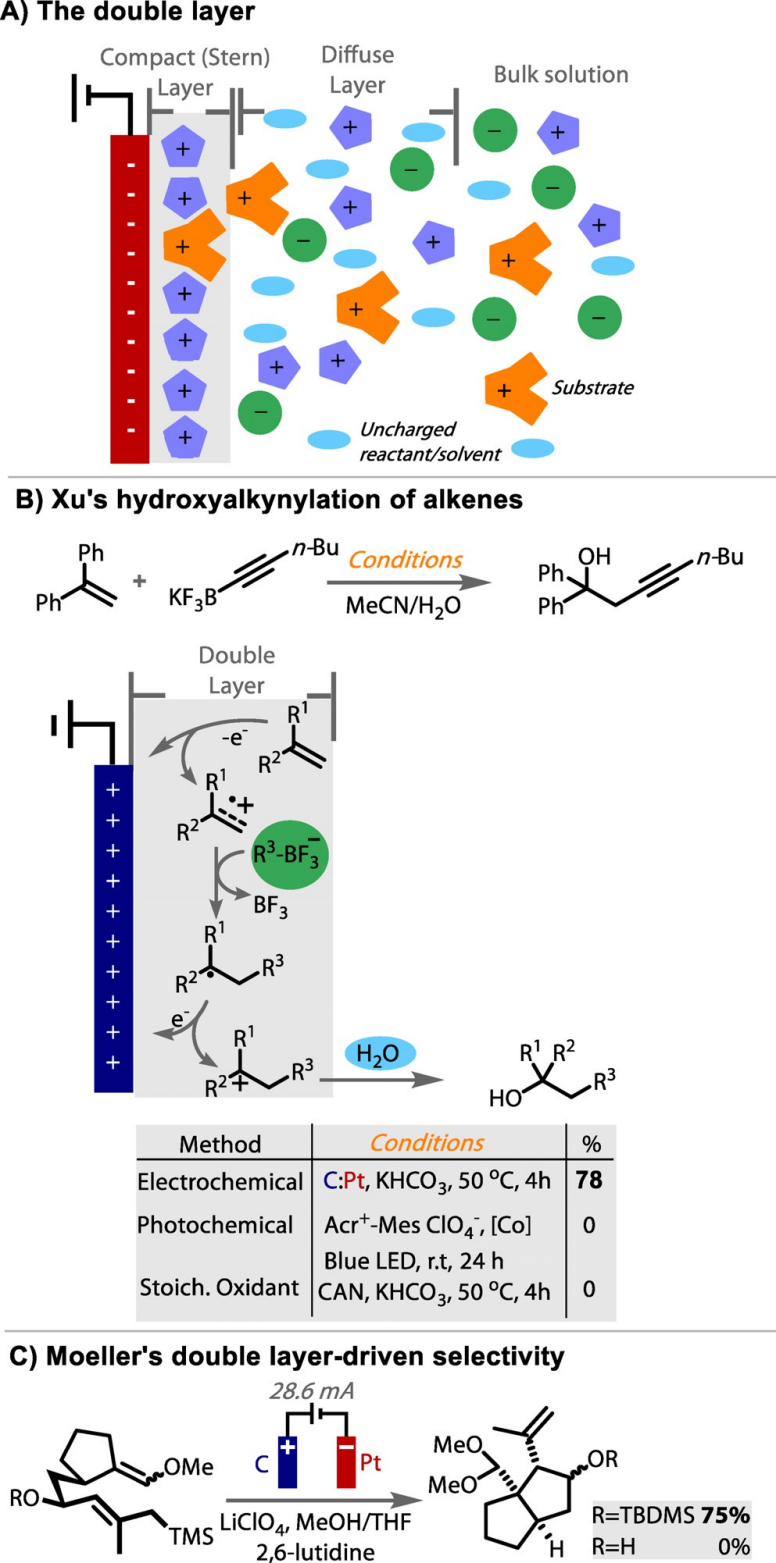
A) Cartoon of the interfacial double layer formed at a cathode; B) double layer‐controlled selectivity of nucleophile attack; C) exclusion of MeOH from double layer promotes cyclisation.

Waldvogel manipulated the interfacial region in the reduction of menthone oximes through the addition of quaternary ammonium salts (Figure 9 B).^119^ These small, hard cationic species form a compact and robust layer on the cathode surface (Figure 15 A). It was found that di‐ or poly‐ammonium salts separated by an alkyl chain also gave superior reaction outcomes, possibly due to an entropic effect. The hard, lipophilic layer was able to exclude both solvent and protons from the surface, and decrease side‐reactions. The ammonium salts serve to attenuate corrosion of the lead cathode, suppressing the formation of lead sulfate deposits to keep a shiny intact surface. The adsorption of ammonium cations also serves to increase the hydrogen overpotential of the cathode by reducing the rate of the HER. This effect was further studied in an amide deoxygenation reaction.^118^ It was proposed that the cationic layer still allows the tunnelling of electrons to reduce substrate, but protons are repelled due to coulombic repulsion. By avoiding competing proton reduction, the double layer protects the electrode from corrosion, and leads to an improved performance. Another example of the use of quaternary ammonium salts to suppress hydrogen evolution was demonstrated by Bhanage in the reduction of *N*‐alkoxyamides to esters.^191^

Reactive species, such as cations or radicals, can readily react with solvent molecules to form undesired products. However, when formed at an electrode within the double layer, solvent can be excluded, which can aid reactivity and enhance selectivity. An elegant example of this is from Xu, who reported an alkynyl‐hydroxylation reaction of alkenes, which is highly regio‐ and chemo‐selective and proposed to only be successful because the selectivity‐determining alkyne addition step occurs within the polarised double layer (Figure 15 B).^192^ The negatively charged alkynyltrifluoroborate nucleophile is attracted to the region and creates a localised high concentration, while competing neutral nucleophiles, such as water, are excluded. Oxidation at an electrode was found to be essential for these reasons, as photochemical or chemical oxidation conditions facilitated direct reaction with water in preference to alkyne.

Moeller also demonstrated that an ordered double layer can improve selectivity in an intramolecular cyclisation reaction by promoting cyclisation over solvent trapping (Figure 15 C).^193^, ^194^, ^195^ The anodic oxidation and the ensuing cyclisation occur within the ordered environment of the double layer at the anode surface, which slows diffusion and excludes the methanol from the electrode surface that could otherwise interfere with cyclisation. Silyl protection of the internal alcohol moiety of the substrate was still necessary to prevent the intramolecular trapping by this competing nucleophile.

## 5 Inert Electrodes

At the other end of the scale to the significant involvement of the electrode and high levels of electrocatalysis is electron transfer from an inert electrode that does not participate in the mechanism and has little substrate or intermediate adsorption. An outer‐sphere‐type electron transfer mechanism is more dominant, which results in high overpotentials for specific reactions. The best‐known inert material is boron doped diamond (BDD), the use of which in organic electrosynthesis has primarily been driven by Waldvogel.^36^, ^196^, ^197^, ^198^, ^199^, ^200^ Although, the level of interaction of an electrode in a reaction is very difficult to determine, BDD has the highest known overpotential for the oxygen and hydrogen evolution reactions, which indicates very low levels of interaction. Because of this, BDD also offers a very high potential window and is highly chemically inert. However, it has been shown that the level of boron doping can actually affect selectivity,^201^ and sp^2^ non‐diamond carbon impurities alters the potential window,^144^ implying that the material is not perfectly inert. BDD has been reported to yield differences in selectivity to other materials in various reactions, such as CO_2_ reduction.^202^, ^203^, ^204^ However, herein, we describe several synthetic organic examples that have required the use of a more inert electrode, which BDD has fulfilled. More specific features of BDD and its general use has been well reviewed elsewhere.^137^, ^205^, ^206^, ^207^

**Figure 16:**
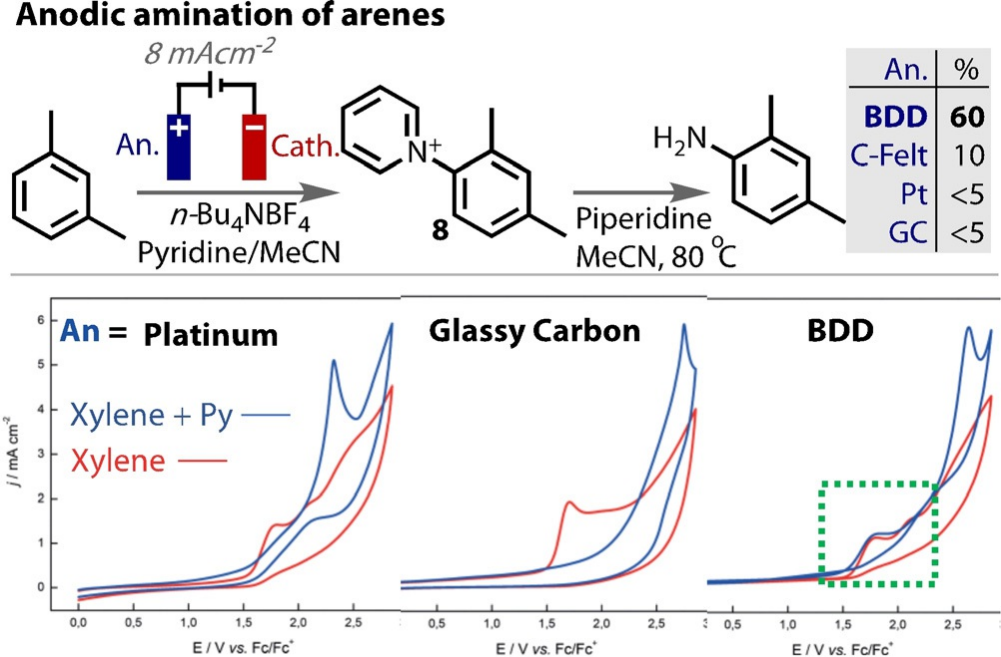
Anodic amination of arenes by Waldvogel showing unique performance of BDD anode. Cyclic voltammograms of *m*‐xylene (red) and *m*‐xylene with pyridine (blue), on platinum, glassy carbon and BDD. Green dashed box highlights the unaffected oxidation feature of xylene upon addition of pyridine on BDD anode.

The electrochemical C−H amination of arenes via Zincke intermediates (**8**) was reported by Yoshida using a carbon felt anode and platinum plate cathode, Figure 16.^208^ However, the scope was limited to electron rich rings containing methoxy groups. In an effort to widen the scope toward arenes with less electron density, Waldvogel explored the use of different anode materials in the reaction.^200^ While retaining the Pt counter cathode in a divided cell set up, the use of carbon felt or fleece anodes were confirmed to give moderate or poor results for the amination of alkylated arenes. These porous carbon materials have high surface area, which causes diffusion of the radical cation away from the electrode to be more difficult, as it is liable to adsorb and oxidise further. Platinum, glassy carbon and isostatic graphite anode materials all returned very poor yields, with electrode fouling observed with the former two and corrosion with the latter. However, switching to a BDD anode resulted in a significant boost in yield, up to 60 %. CV studies were conducted to gain greater insight into the enhanced performance of BDD compared to platinum and glassy carbon. CV traces of xylene (red) and xylene with pyridine (blue) were recorded and compared (Figure 16). With the use of Pt and glassy carbon anodes, the oxidation feature of xylene disappeared upon addition of pyridine. However, with a BBD anode the CV trace was unaffected, and the oxidation of xylene was retained. This trend correlates with the outcome of the amination reaction at each anode material.

**Figure 17:**
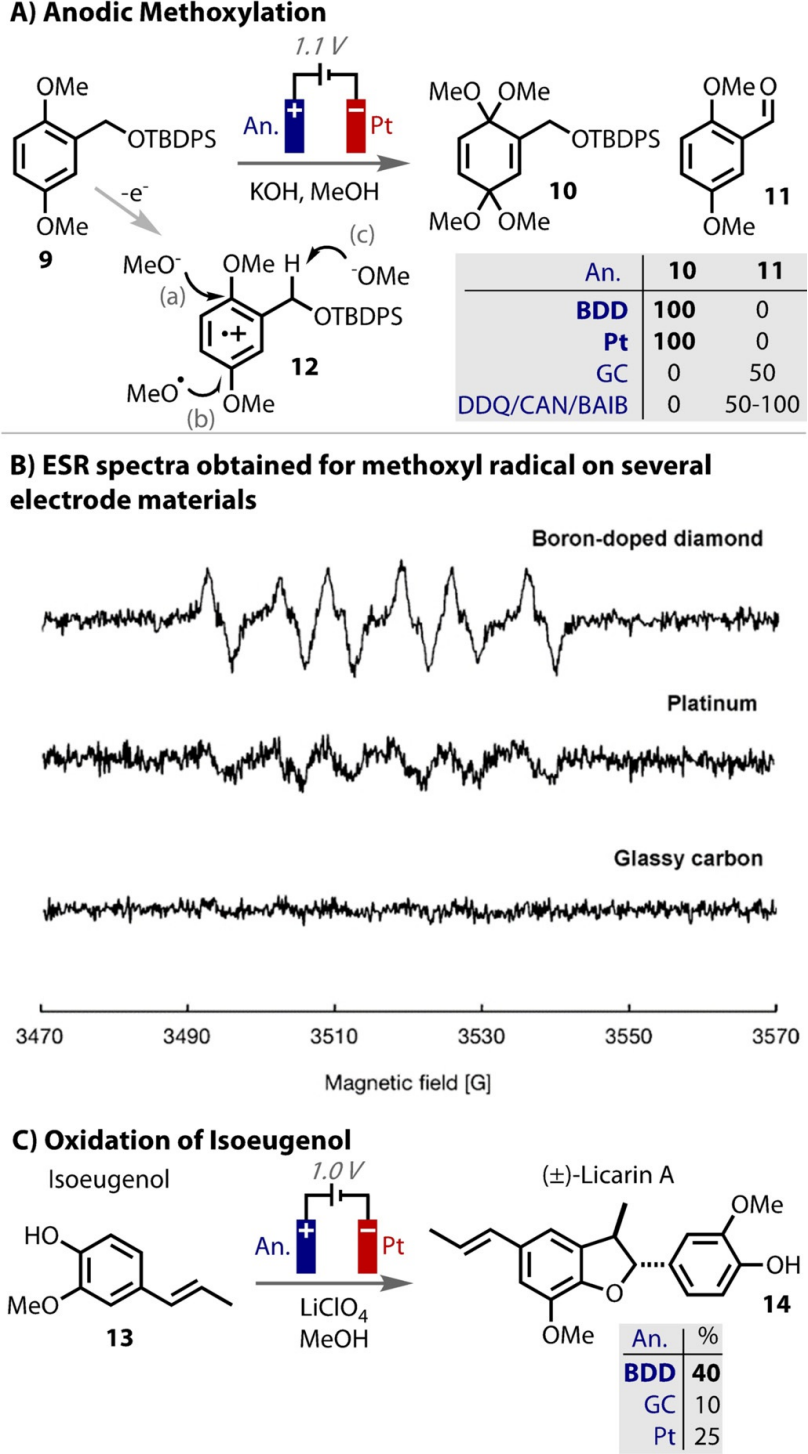
A) Two possible mechanisms for the oxidative methoxylation of **9** (a) and (b) to give **10**. Aldehyde **11** is formed from (c); B) ESR spectra reveal methoxyl radicals, leading to mechanism (b); C) formation of methoxyl radicals are more efficient on BDD anode in oxidation of isoeugenol.

An electrochemical dimethoxylation was a key step in Nishiyama and Einaga's synthesis of (±)‐parasitenone.^209^, ^210^ The use of BDD and Pt for the oxidation of **9** gave excellent yields of the desired product **10**. However, glassy carbon or the use of chemical oxidants returned a different, aldehyde product **11** (Figure 17 A). Anodic oxidation of **9** leads to the radical cation **12**, from which **11** is formed from methoxide deprotonating the benzylic position (via route *c*). Although methoxide attack of the ring leads to the product **10** (via route *a*), the anode material dependency on the reaction selectivity suggests an alternative electrode material‐dependent mechanism. ESR studies revealed the formation of methoxyl radicals, most efficiency with a BDD anode, to a lesser extent with Pt, but not at all with GC (Figure 17 B). The authors proposed that these data signal that methoxyl radical attack onto the radical cation is the leading pathway to product **10** (via route *b*) and were used to explain the selectivity differences observed with each anode material. As methoxyl radicals are highly reactive, an inert electrode proved to be essential for this transformation.

The use of a BDD anode led to the highest yields in the challenging oxidation of isoeugenol (**13**) to (±)‐Licarin A (**14**) (Figure 17 C).^210^ This was similarly proposed to be due to the more efficient formation of methoxyl radicals on BDD. Lower yields of the desired product **14** and overall mass balance were observed with Pt and lower still with glassy carbon. The other side‐products that also required the formation of highly reactive radicals were also formed in greater quantities with BDD. Interestingly, the oxidation of isoeugenol on BDD in hexafluoro isopropanol (HFIP) give the homo‐coupled product, diisoeugenol.^211^

**Figure 18:**
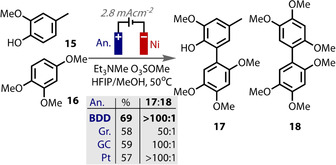
BDD gives best yields for phenol/arene coupling reaction, which produces reactive radical intermediates.

Waldvogel tested the influence of anode materials in the cross‐coupling of phenols and arenes. Preliminary studies revealed that the use of carbon electrodes gave only homo‐coupled adducts.^196^ Platinum plates improved the yield and selectivity of cross to homo‐coupled ratio to 1:1, but a switch to BDD gave a further enhancement in the selectivity (1.5:1). Further optimisation led to a set of improved conditions that contained methanol or water as an additive (Figure 18).^212^ When the electrode material was varied again, BDD, Pt and GC all gave excellent selectivity, but BDD proved to be the superior anode material with respect to yield. These observations led the authors to propose the formation of alkoxyl radicals, stabilised by the HFIP environment and which mediated the generation of the phenoxyl radical intermediate. Further studies revealed the alcohol additive beneficially altered the oxidation potentials of the substrates.^213^ Nonetheless, the use of BDD as an inert electrode had a positive effect on the outcome of the reaction.

## 6 Sacrificial Electrode

The use of a metal anode with a very low oxidation potential will leech metal ions into the solution upon its oxidation. In this case, it is termed a sacrificial electrode, as it is being consumed stoichiometrically. This approach is practical, facile and so frequently applied as a counter electrode process in electrochemical reduction reactions. Common choices for a sacrificial anode include zinc, magnesium or aluminium. In many cases, the choice is dictated by price or toxicity concerns, with little effect on the reaction observed. However, commonly, the liberated ions play a role in the reaction by coordinating reactants or products, and maintaining high conductivity. Care should be taken to avoid reduction of the liberated ions on the cathode to avoid short circuiting the system, hence the use of highly reducing metals that thermodynamically disfavour this process.

**Figure 19:**
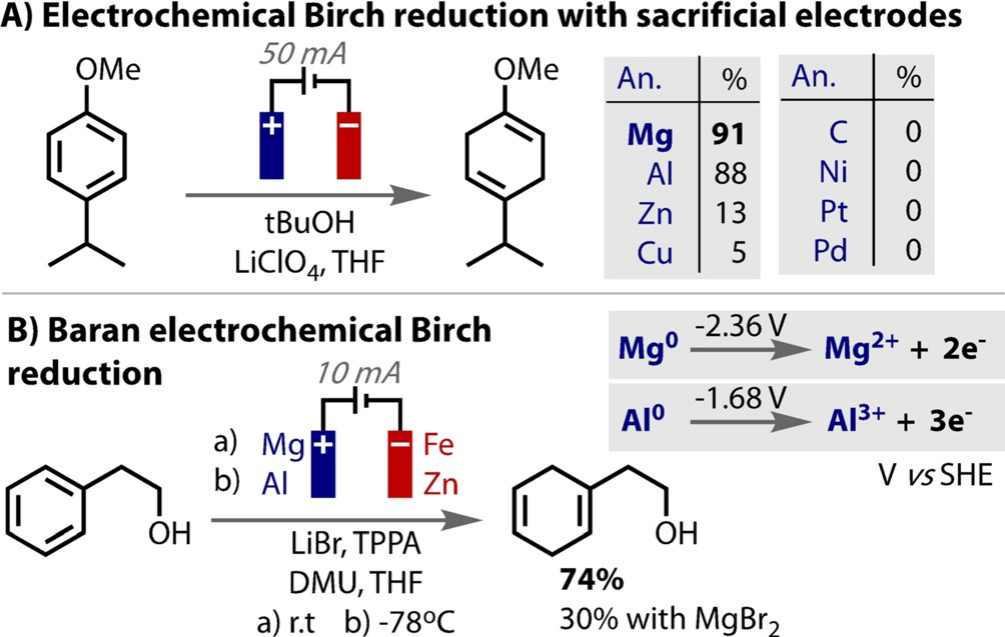
A) Mg as sacrificial electrode leads to most efficient reactivity; B) Mg or Al as sacrificial electrode. Mg^2+^ proposed not to play significant role in mechanism.

The reduction of organohalides is a reaction in which a sacrificial anode is frequently used,^214^, ^215^ in particular for carboxylation reactions by coupling with CO_2_ as an electrophile.^57^, ^139^, ^216^, ^217^, ^218^, ^219^, ^220^ The metal ions liberated from the anode stabilise the carboxylate product, which also helps to prevent anodic Kolbe‐type reactions of the carboxylate.^57^, ^165^, ^220^ A more recent example is the use of either Mg or Al anodes by Baran in an electrochemical Birch reduction (Figure 19 B). The reaction was shown to be highly scalable and remarkably tolerant to a very wide range of substrates.^91^ In an earlier study of the same reaction (Figure 19 A),^86^ Mg anodes were also found to give the highest yields, which was proposed to be due to Mg^2+^ ions acting as electron transfer catalysts or stabilising anionic intermediates and promoting their reduction. Mechanistic studies were undertaken by Baran to elucidate if liberated Mg^2+^ ions played a beneficial role in the reaction. The addition of MgBr_2_ as an additive only served to decrease the product yield, although it seems likely that its reduction may compete with substrate reduction. In the absence of stirring, diminished yields could also be correlated to smaller electrode separation distances. These data suggest the diffusion of metal ions to the anode is deleterious to the reaction and so the liberated ions are not mechanistically relevant.

**Figure 20:**
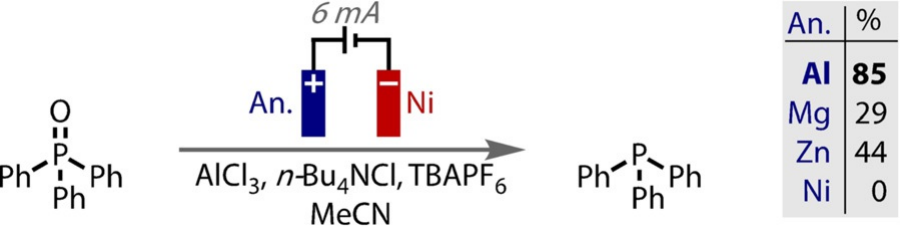
Phosphine oxide deoxygenation is aided by sacrificial Al anode for in situ Lewis acid generation.

Rather than a sacrificial anode producing only waste products, it can be used to liberate reagents into solution in a controlled manner that matches reaction progress, often yielding results that are not possible by other means. In an elegant recent example of this, Sevov reported the use of an aluminum anode in the deoxygenation of phosphine oxides (Figure 20).^221^ The sacrificial electrode oxidises to liberate aluminum ions into solution that combine with chloride ions to form an amine‐stabilised AlCl_3_ complex. This in situ generated Lewis acid activates the phosphine oxide, producing a less negative potential for its reduction and subsequent deoxygenation. As 2 electrons are required for the phosphine oxide reduction, and 3 are removed from Al^0^ to give Al^3+^, an additional quantity of added AlCl_3_ is required to balance the stoichiometry and ensure high reaction efficiency, without which, a lower performance was observed.

**Figure 21:**
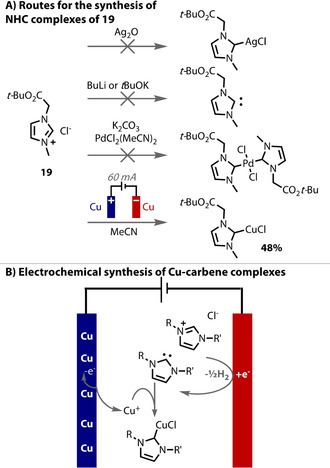
A) NHC complexes generated from an electrochemical process; B) the mechanism for their formation.

Metal anodes have been employed as sacrificial electrodes by Willans to provide metal ions into solution, at a controlled rate and with control of the oxidation state, in order to generate organometallic complexes that are not otherwise obtainable (Figure 21 A). Examples of this approach include the use of copper, iron and manganese anodes in the presence of NHC and salen ligands, to form the corresponding complexes.^222^, ^223^, ^224^ The reaction is a paired process (Figure 21 B), that is, the ligand precursor is reduced on the cathode and the metal ions are produced on the anode. The procedures are remarkably straightforward and yield high purity complexes without the necessity for column chromatography. Similarly, Mellah demonstrated Sm^II^ complexes could be efficiently prepared through the use of a sacrificial samarium anode, and are important catalysts.^225^, ^226^, ^227^

## 7 Summary and Outlook

While a rational choice of electrode material for use in organic electrochemical transformations cannot yet be made readily and reliably, herein, we have highlighted where efforts have moved beyond screening and empirical investigations. In many cases, efforts to understand the influence of electrode material through analytical electrochemistry and a physical organic chemistry approach have led to the elucidation of trends. Such trends and insight may be applied more broadly, which will lead to enhanced efficiencies and new opportunities. Due to the complexities and variation of electrode‐substrate interactions in organic transformations, it is likely that experimentation will remain necessary, even when the choice is guided by principles. To aid exploration of the breadth of materials available, two tables are appended summarising key materials, their properties, and applications in electrosynthesis (Tables 2 and 3).

**Table 2:** A summary of the key properties, forms, sources and example uses of common electrode materials used in organic electrosynthesis. Pricing information obtained January 2020.

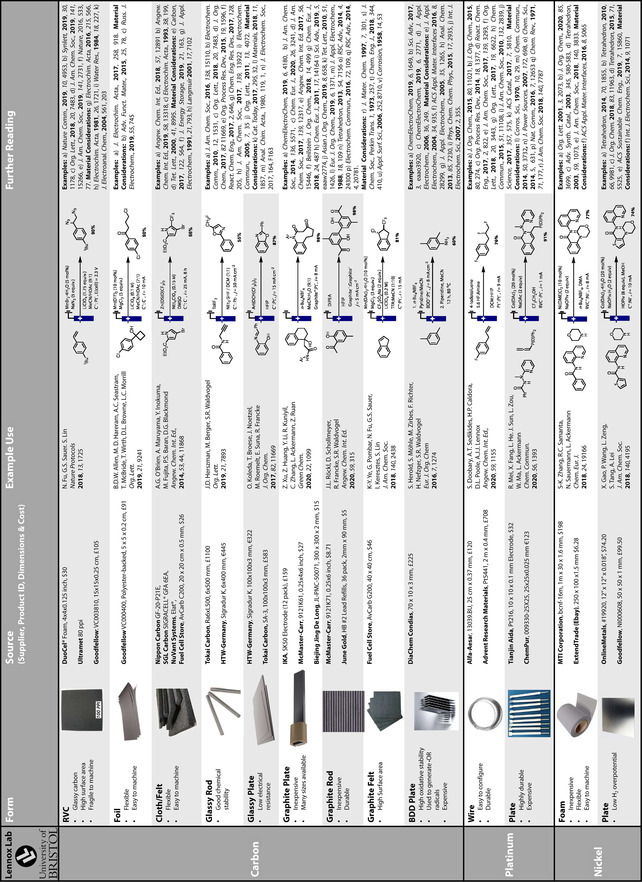

**Table 3:** A summary of the key properties and example uses of electrode materials for organic electrosynthesis.

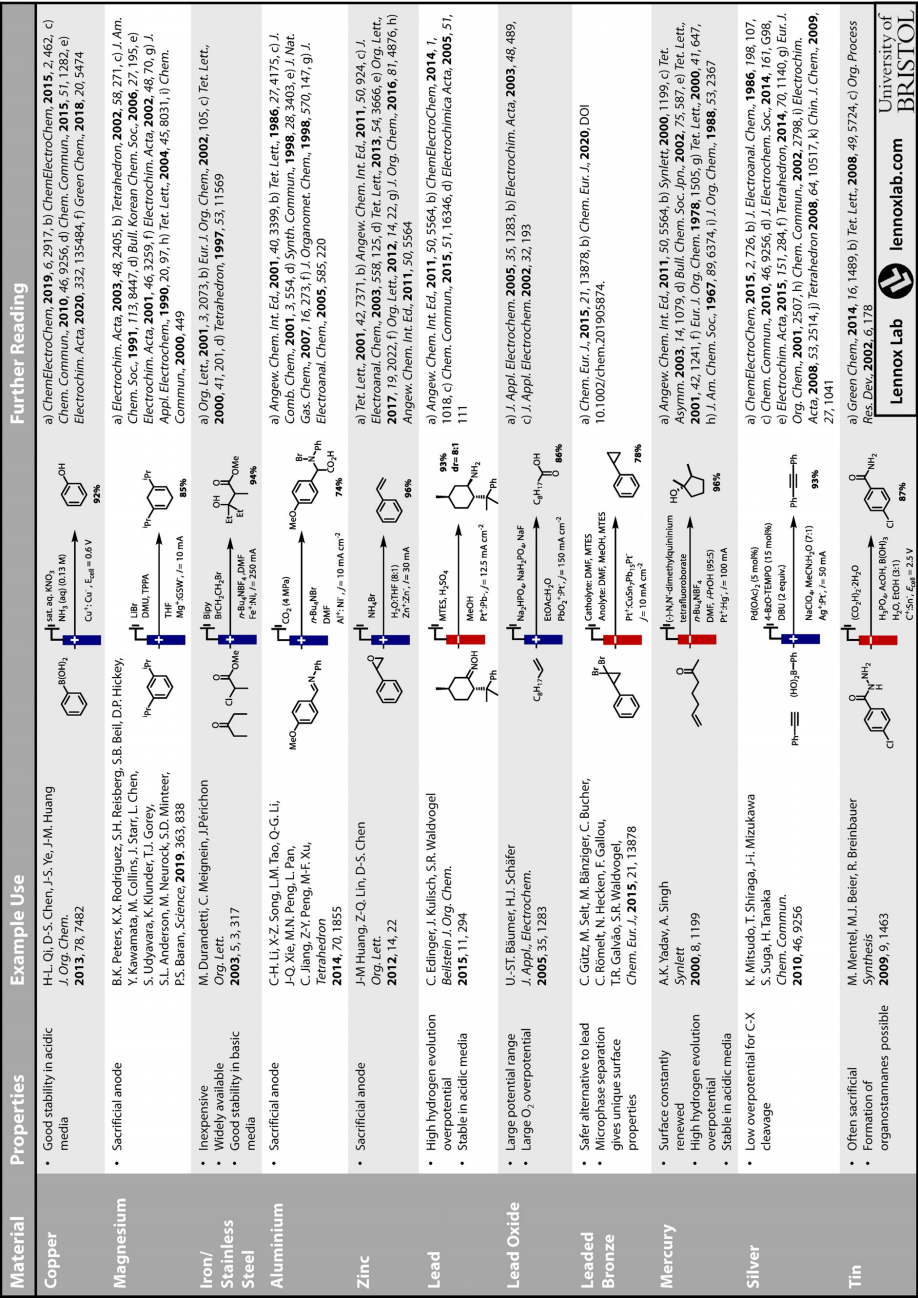

The criteria for an ideal electrode material is that it is inexpensive, non‐toxic, stable, manipulatable, resist corrosion and, most importantly, provide high yields and exquisite selectivity. While a number of materials perform extremely well and fit many of these criteria, it is clear that there is currently no material that meets all of them. These criteria are also reaction‐specific, as cost, selectivity and yield have to be balanced against the cost of product and ease of access to it by other means. The development of new materials and the design of robust electrocatalysts for organic synthesis also still lags many other applications of electrochemistry, yet may provide new opportunities for the field. While the electrode material remains a key optimisation parameter, it holds great opportunity to impart new reactivity and greater reaction efficiency.

## Conflict of interest

The authors declare no conflict of interest.

## Biographical Information

*David Heard studied at the University of Sheffield (MChem, 2014) then joined the Bristol Chemical Synthesis CDT at the University of Bristol. He conducted his PhD research into the structural elucidation and total synthesis of maleidride natural products under the supervision of Prof. Chris Willis. David has been a member of the Lennox group since 2019, and is investigating new methods for electrochemical reaction optimisation*.

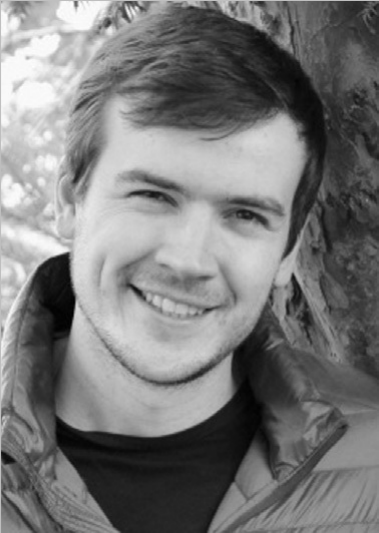

## Biographical Information

*Alastair Lennox is a graduate of the University of Manchester and completed his PhD in 2012 under the supervision of Professor Guy Lloyd‐Jones. Following postdoctoral research with Prof. Matthias Beller as an Alexander von Humboldt Fellow, and with Prof. Shannon Stahl at the University of Wisconsin‐Madison, he joined the University of Bristol as a Royal Society University Research Fellow. His research group's interests include the development of new selective and sustainable electrochemical methodologies for pharmaceutical and agrochemical use*.